# Digestive symptoms in daily life of chronic adrenal insufficiency patients are similar to irritable bowel syndrome symptoms

**DOI:** 10.1038/s41598-021-87158-2

**Published:** 2021-04-13

**Authors:** L. Quénéhervé, D. Drui, J. Blin, M. Péré, E. Coron, G. Barbara, M. R. Barbaro, B. Cariou, M. Neunlist, D. Masson, K. Bach-Ngohou

**Affiliations:** 1 INSERM, TENS, The Enteric Nervous System in Gut and Brain Diseases, IMAD, University of Nantes, Nantes, France; 2 Institut des Maladies de l’Appareil Digestif, IMAD, CHU Nantes, Hôpital Hôtel-Dieu, Nantes, France; 3 Department of Endocrinology, l’Institut du Thorax, CHU Nantes, 44400 Nantes, France; 4 Department of Biology, Laboratory of Clinical Biochemistry, CHU Nantes, 9 Quai Moncousu, 44000 Nantes, France; 5 Biostatistics Unit, Research Board, CHU Nantes, Nantes, France; 6 Department of Medical and Surgical Sciences, Centre for Applied Biomedical Research, University of Bologna, IRCCS S. Orsola, Bologna, Italy; 7 Department of Endocrinology, CNRS, INSERM, l’Institut du Thorax, CHU Nantes, Université de Nantes, 44400 Nantes, France

**Keywords:** Endocrine system and metabolic diseases, Adrenal gland diseases, Functional gastrointestinal disorders, Irritable bowel syndrome

## Abstract

Gastrointestinal symptoms are frequent in acute adrenal insufficiency. Although digestive symptoms can significantly reduce quality of life, they are rarely described in patients with treated chronic adrenal insufficiency (CAI). We aimed to characterize digestive symptoms in CAI patients. We used the section pertaining functional bowel disorders of the Rome IV questionnaire. A questionnaire was published on the website of the non-profit patient association “Adrenals” (NPPA of CAI patients) for five months. Information on demographics, characteristics of adrenal insufficiency, digestive symptoms and quality of life was collected. The relatives of CAI patients served as a control group. We analyzed responses of 33 control subjects and 119 patients (68 primary adrenal insufficiency (PAI), 30 secondary adrenal insufficiency (SAI) and 21 congenital adrenal hyperplasia (CAH)). Abdominal pain at least once a week over the past 3 months was reported by 40%, 47% and 33% of patients with PAI, SAI and CAH respectively versus 15% for the controls (*p* = 0.01). Symptoms were consistent with the Rome IV criteria for irritable bowel syndrome in 27%, 33% and 33% of patients respectively versus 6% for the controls (*p* < 0.0001). Quality of life was described as poor or very poor in 35%, 57% and 24% of patients respectively versus 5% for the controls (*p* < 0.0001). In conclusion, digestive symptoms are frequent and incapacitating in CAI patients and similar to symptoms of irritable bowel syndrome in 30% of CAI patients. Assessment and management of digestive symptoms should be considered a priority for physicians treating patients with CAI.

## Introduction

CAI results either from PAI, which is rare with a prevalence of 82–144/million in Europe, or from SAI, with a prevalence twice as high^1^. Primary adrenal insufficiency occurs after destruction of the adrenal glands (e.g., due to auto-immune disease, infection, hemorrhage, cancer, bilateral adrenalectomy, etc.), or is caused by metabolic failure in hormone production, the more frequent condition being CAH resulting from genetic 21-hydroxylase deficiency. SAI, results from the impairment of the hypothalamic–pituitary–adrenal axis due to Adreno Cortico Tropic Hormone (ACTH) secretion deficiency.

Digestive symptoms (abdominal pain, nausea, vomiting) have been described as some of the many non-specific manifestations of acute and CAI prior to diagnosis, and sometimes lead to misdiagnosis of an acute abdomen. They are also reported in cases of adrenal crisis, a life-threatening complication of CAI, resulting from acute glucocorticoid deficiency^2, 3^. Digestive manifestations are thought to be a direct consequence of glucocorticoid and mineralocorticoid deficiency^4^, however their mechanisms are not well known. Digestive symptoms tend to decrease under replacement therapy, as shown in a 426 patients series with autoimmune PAI^5^. However, gastrointestinal symptoms are frequently reported in patient NPPA surveys^6^.

In spite of replacement therapy, studies have highlighted an impairment of the health-related quality of life (HRQoL) in patients with CAI^7–9^, using nonspecific scores, that do not explore gastrointestinal symptoms in depth. This impairment can be explained, at least in part, by the limits of medication, which can only mimic physiological steroid secretion, without its circadian variations. Thus, a better understanding of treatment effects is key to improve the HRQoL of these patients^10^. HRQoL is an individual’s perceived physical and mental health. This questionnaire explores four core questions about health in general, physical health (which includes physical illness and injury), mental health (which includes stress, depression, and problems with emotions), ability to perform usual activities (such as self-care, work, or recreation) and ten additional questions about health-related quality of life (about recent pain, depression, anxiety, sleeplessness, vitality, and the cause, duration, and severity of a current activity limitation). This questionnaire permits to highlight health disparities between different groups of patients who present different pathologies. In endocrine disorders, disease-specific HRQoL questionnaires have proven useful for clinical research and follow-up of patients but the specific score for adrenal insufficiency, AddiQol^6^ contains only one question about gastrointestinal (GI) symptoms (“I get nauseous”) which does not reflect abdominal discomfort nor altered bowel habit.

The prevalence of digestive symptoms in cases of apparently balanced CAI is not well known. The objective of this study was to evaluate the prevalence of digestive symptoms in a real-life population of patients with treated adrenal insufficiency and to appreciate their overall quality of life.

## Results

### Population studied

**Figure 1:**
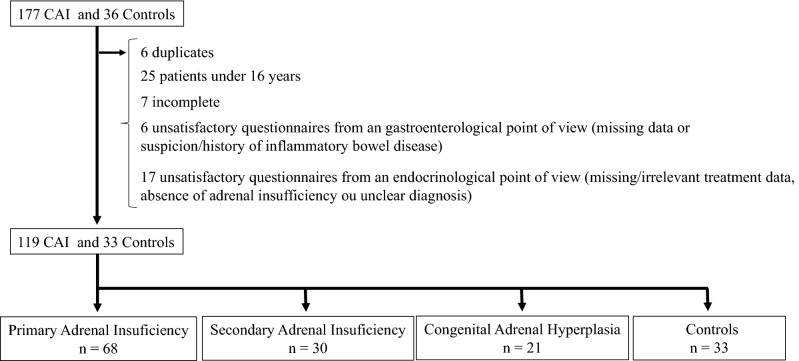
Flow chart for the population studied. *CAI* chronic adrenal insufficiency.

Of the 177 questionnaires of CAI patients, 6 were duplicates, 25 were from patients under 16 years old, and 6 were incomplete. After review by a gastroenterologist, 4 patients were excluded because of missing data or suspicion/history of inflammatory bowel disease. After review by an endocrinologist, 17 patients were excluded because of missing/irrelevant treatment data, absence of adrenal insufficiency or unclear diagnosis. Of the 36 questionnaires of controls, 1 was incomplete, 2 were excluded respectively because of history of inflammatory bowel disease and chronic treatment by glucocorticoid for asthma. Flow chart for patients and controls is presented in Fig. 1.

Responses from 119 patients and 33 controls were analyzed. Among patients, 68 (57%) had PAI, 30 (25%) had SAI and 21 (18%) had CAH (Table 1). Strong female predominance was observed in the CAI group with 101 women (85%), homogenous between the PAI, SAI and CAH groups with 84%, 90% and 81% respectively, compared to 16 women (48%) in the control group (*p* = 0.0004). Mean age was 45 years [17–74] in the CAI group versus 46 years [30–70] in the control group. All CAI patients were treated with hydrocortisone, 81 (68%) with fludrocortisone (65 (96%), 1 (3%) and 16 (76%) in PAI, SAI and CAH groups respectively), 8 (6.7%) with dehydroepiandrosterone and 3 (2.5%) with dexamethasone. 89 patients (75%) were considered as having an appropriate substitution treatment, 10 (8%) underdosed and 20 (17%) overdosed. Two patients of our cohort reported an history of celiac disease.

**Table 1:** Patient characteristics.

	PAI (n = 68)	SAI (n = 30)	CAH (n = 21)	Total (n = 119)
Age, years mean ± SD	48 ± 13	46 ± 12	35 ± 12	45 ± 13
**Sex**				
Female	57 (84%)	27 (90%)	17 (81%)	101 (85%)
Male	11 (16%)	3 (10%)	4 (19%)	18 (15%)
Age at diagnosis (years) mean [min; max]	36 [11;67]	36 [8;61]	0* [0;27]	35 [5;67]
Hydrocortisone tt, n (%)	68 (100%)	30 (100%)	21 (100%)	119 (100%)
Dose(mg/j) mean ± SD	25 ± 6	23 ± 5	24 ± 9	24 ± 7
Fludrocortisone tt, n (%)	65 (96%)	1 (3%)	16 (76%)	82 (69%)
Dose(mg/j) mean ± SD	80 ± 42	50 ± 0	79 ± 50	80 ± 43
Dehydroepiandrosterone tt, n (%)	7 (10%)	1 (3%)	0 (0%)	8 (7%)
Dexamethasone tt n (%)	1 (1%)	0 (0%)	2 (10%)	3 (3%)
Levothyroxine tt n (%)	14 (21%)	7 (23%)	0 (0%)	21 (18%)
Other HRT n (%)	1 (1%)	5 (17%)	1 (5%)	7 (6%)

*PAI* primary adrenal insufficiency, *SAI* secondary adrenal insufficiency, *CAH* congenital adrenal hyperplasia, *tt* treatment, *HRT* hormone replacement therapy.

*At birth in 16 patients.

Twenty-one patients reported a replacement therapy of l-Thyroxin, 14 (21%) in the PAI group and 7 (23%) in the SAI group, without significant difference between the 2 groups. In the control group, one patient (3%) was treated with replacement therapy of l-Thyroxin (*p* = 0.006). Finally, 7 patients were treated for hormone deficiency other than hypothyroidism, which was in most cases gonadotropic deficiency, mainly in the SAI group with 5 patients compared with 1 patient in both PAI and CAH groups. None of the control subjects were treated for other hormone deficiency.

### Digestive assessment and quality of life

Fifty-four CAI patients (46%) claimed to suffer from a digestive disease; among them 17 (31%) stated that the digestive disease existed prior to the diagnosis of CAI and 37 (69%) that it did not exist at the time of diagnosis. CAI patients reported gastroenteritis in 44 cases (38%), with a mean of 3.7 ± 7.6 episodes/year. CAI patients had a known history of IBS in 21 cases (18%).

**Figure 2:**
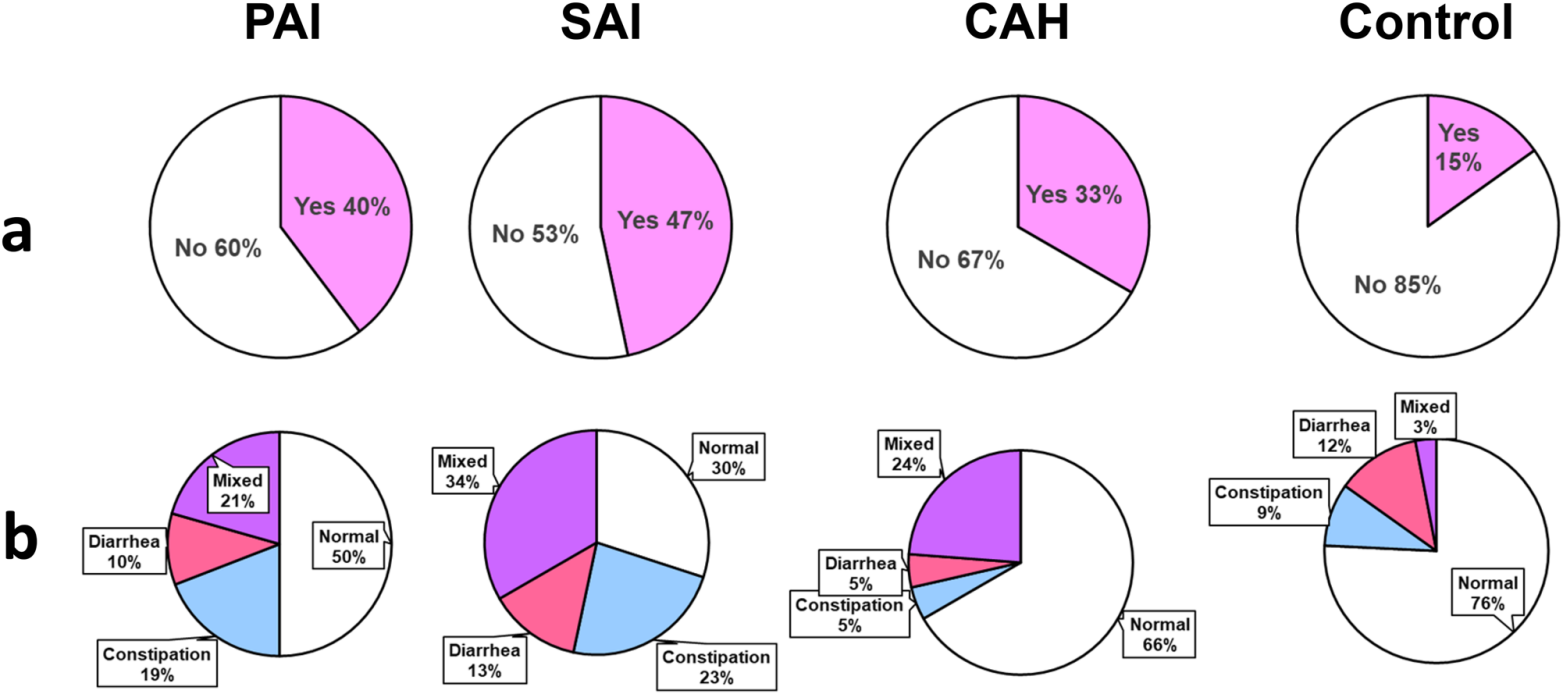
Digestive symptoms reported by patients in each adrenal insufficiency subgroup and control group. (**a**) Percentage of patients with abdominal pain at least once a week during the last three months (pink: yes (abdominal pain), white: no abdominal pain); (**b**) Type of bowel habit according to the Bristol stool scale (normal: white, constipation: blue, diarrhea: red or mixed: purple). *PAI* primary adrenal insufficiency, *SAI* secondary adrenal insufficiency, *CAH* congenital adrenal hyperplasia.

Forty-eight CAI patients (40%) reported abdominal pain at least once a week during the last three months without any statistical difference between the PAI, SAI and CAH with 27 (40%), 14 (47%) and 7(33%) patients respectively (*p* = 0.63), compared to 5 in the control group (15%) (*p* = 0.01) (Fig. 2a).

Sixty-two CAI patients (52%) had abnormal bowel habits according to the Bristol stool scale, with 34 (50%), 21 (70%) and 7 (33%) in the PAI, SAI and CAH groups respectively, compared to 8 (24%) in the control group (*p* = 0.009) (Fig. 2b). Mixed bowel habits with, alternatively, constipation and diarrhea were the most frequent disorder with 29 patients (24%), then constipation 21 (18%) and diarrhea in 12 (10%), without any statistical difference between groups.

History of IBS was the only detected risk factor for abdominal pain in univariate (Table 2) and multivariate analysis (OR: 7.04 [2.36; 21.00] *p* = 0.0005).

**Table 2:** Risk factors for abdominal pain (univariate analysis).

Variable	N	OR	IC 95%		*p* value
Sex: Male	119	0.52	[0.17; 1.57]	0.2443	
**Age**	**119**	**0.97**	**[0.95; 1.00]**	**0.0692**	
Cause of chronic adrenal insufficiency	119				
SAI versus PAI		1.33	[0.56; 3.16]	0.5202	
CAH versus PAI		0.76	[0.27; 2.12]	0.5999	
Duration of chronic adrenal insufficiency	103	1.00	[0.97; 1.04]	0.8013	
**Diagnosed irritable bowel syndrome**	**117**	**7.04**	**[2.36; 21.00]**	**0.0005**	
Dexamethasone (treatment)	119	0.73	[0.06; 8.33]	0.8030	
Fludrocortisone (treatment)	119	0.61	[0.28; 1.34]	0.2161	
Dehydroepiandrosterone (treatment)	119	1.52	[0.36; 6.41]	0.5663	
Levothyroxine (treatment)	119	0.70	[0.26; 1.87]	0.4725	
Associated hormonal deficiency	117	0.34	[0.04; 3.11]	0.3366	
Associated hormonal deficiency (other than hypothyroidism)	119	0.23	[0.03; 1.98]	0.1810	

Bold values represent variables with *p*-value < 0.2 that are included in the construction of the multivariate model.

*SAI* secondary adrenal insufficiency, *PAI* primary adrenal insufficiency, *CAH* congenital adrenal hyperplasia.

**Figure 3:**
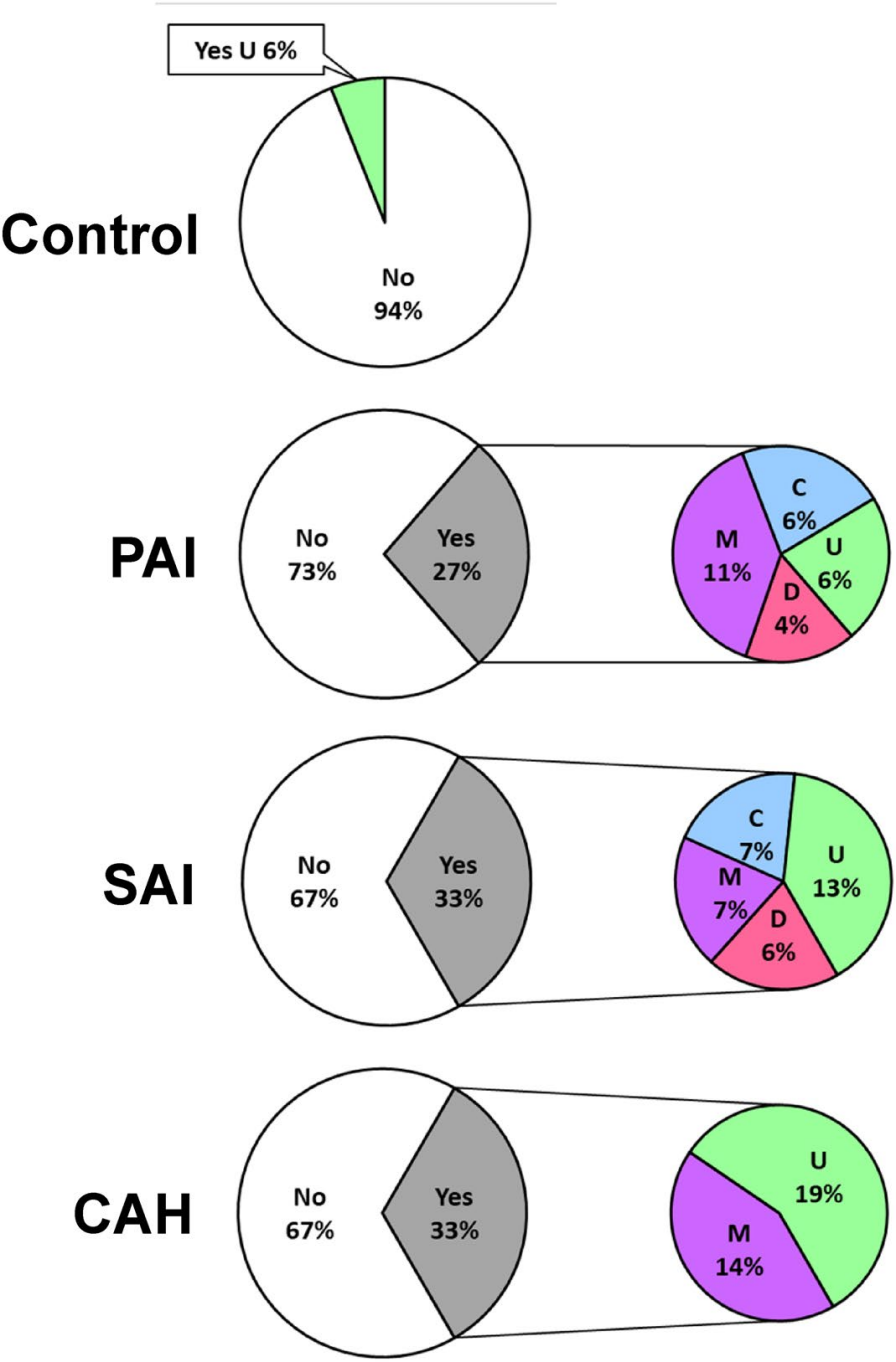
Percentage of patients fulfilling Rome IV criteria for the diagnosis of irritable bowel syndrome. Left pie chart—white: no (no IBS criteria), grey: yes (IBS criteria). The two control subjects fulfilling IBS criteria were both unsubtyped IBS (Green: U); right pie chart (IBS subtypes)—red: diarrhea IBS criteria (D), blue: constipation IBS criteria (C), purple: mixed bowel habits IBS criteria (M) and green: unsubtyped IBS (U). *IBS* irritable bowel syndrome, *PAI* primary adrenal insufficiency, *SAI* secondary adrenal insufficiency, *CAH* congenital adrenal hyperplasia.

Symptoms were consistent with the Rome IV IBS criteria in 35 (30%) of CAI patients, with 18 (27%), 10 (33%) and 7 (33%) of patients with PAI, SAI and CAH respectively, without any statistical difference between groups (*p* = 0.78) (Fig. 3). IBS-like symptoms were significantly more frequent in CAI patients than in controls (n = 2, 6%) (*p* ≤ 0.0001). Regarding IBS subtypes, 14% of patients fulfilling IBS criteria had diarrhea predominant IBS (IBS-D) criteria, 17% constipation predominant IBS (IBS-C) criteria, 34% mixed bowel habits IBS (IBS-M) criteria and 34% undetermined or unsubtyped IBS (IBS-U). The two control subjects fulfilling IBS criteria were both IBS-U (Fig. 3).

CAI patients were divided in two subgroups depending on Hydrocortisone dose (appropriate and inappropriate treatment). Abdominal pain and IBS-like symptoms were significantly higher in CAI patients than in controls, whatever Hydrocortisone dose (Table 3). CAI patients with inappropriate treatment had digestive symptoms slightly more frequently than those with appropriate treatment (53% vs 36% respectively for abdominal pain, and 43% vs 25% respectively for IBS symptoms) (*p* = 0.09 and *p* = 0.06 respectively) (Table 3).

**Table 3:** Abdominal pain, irritable bowel disease syndrome and hydrocortisone dose.

n	Appropriate treatment	Inappropriate treatment	Controls	*p* value^a^
	89	30	33	
**3A—percentage of digestive symptoms between groups**				
Abdominal pain*	32 (36%)	16 (53%)	5 (15%)	0.01
IBS criteria**	22 (25%)	13 (43%)	2 (6%)	0.008

	Appropriate treatment versus controls	Inappropriate treatment versus control	Appropriate versus inappropriate treatment	
	*p* value^a^	*p* value^a^	*p* value^a^	
**3B—statistical analysis between groups**				
Abdominal pain*	0.05	0.005	0.09	
IBS criteria**	0.04	0.002	0.06	

*Number of subjects with abdominal pain at least once a week during the last 3 months.

**Number of patients fulfilling ROME IV criteria for irritable bowel disease.

A *p* value < 0.05 was considered to be significant.

^a^Chi^2^ test with Holm correction.

Forty-six CAI patients (39%) described their quality of life as poor or very poor (24 (35%), 17 (57%) and 5 (24%) of patients with PAI, SAI and CAH respectively), compared to 5 (15%) of controls (*p* < 0.0001).

## Discussion

In this cross-sectional controlled study of 119 patients with CAI, the prevalence of GI symptoms was higher than in the control group, with 52% of CAI patients reporting abnormal bowel habits and 40% reporting frequent abdominal pain (at least once a week) versus 24% and 15% of controls respectively. Reports of GI symptoms were comparable in different types of adrenal insufficiency.

This exploratory study is the first to our knowledge to specifically address the issue of digestive symptoms in CAI apart from adrenal crisis. As very few studies specifically explore gastrointestinal symptoms in CAI^11^ and as disorders of the upper digestive tract are described more than the lower digestive tract, the results of our study shed new light on these sometimes disabling symptoms that patients do not spontaneously report to their specialist physician.

While most studies focus on patients followed in tertiary centers, our study included patients through a questionnaire published on the website of a patient NPPA. Thus, it allowed us to reach patients with rare conditions, such as CAH, with a significant sample size. However, the fact that patients were answering an online questionnaire, could have led to a sample selection bias. Patients complaining of digestive troubles could have been more prone to answer this questionnaire. However, 24% of patients from this NPPA answered the questionnaire. The characteristics of patients included in this study are comparable to those of the NPPA. PAI, SAI and CAH were reported by 40%, 23% and 37% of the members respectively in 2017, which is comparable to our study. We also have a strong female predominance in CAI groups as compared to controls. Nevertheless, this sex ratio was those observed within the NPPA, with 77% females in the PAI group, 91% in the SAI group and 82% in the CAH group. The higher prevalence of female in our patient group is not unexpected. This higher prevalence is also found in IBS and gender affects gene expression including glucocorticoid receptor expression^12^.

However, the prevalence we have found must be approached with caution. Indeed, as our work was based on an online questionnaire published on the website of a non-profit patient association, our results may not totally reflect GI symptoms of all CAI patients. Thus, even if our study gives an idea of the burden of GI disorders in patients suffering from CAI, our results need to be confirmed by other case–control studies analyzing the digestive symptoms of these patients.

In order to exclude symptoms related to potential frequent adrenal crises, as the current status regarding adrenal balance of patients was unknown, the questionnaire asked specifically for the duration and frequency of abdominal pain. Thus, the analysis focused on chronic symptoms and 59% of patients reported that the abdominal pain lasted for 6 months at least.

The high prevalence of digestive symptoms in the study population could have several explanations. Firstly, patients with CAI are more prone to declare comorbidities, which can be responsible for digestive symptoms, such as celiac disease, which has an increased prevalence in patients with autoimmune etiologies of CAI and should be screened for, in case of digestive symptoms^13^. In this study, 2 patients reported a history of celiac disease. We did not have any information about their gluten consumption, which can lead to GI disorders. However, none of these patients reported neither abdominal pain, nor IBS-like symptoms according to ROME IV questionnaire. Hypothyroidism is also frequent in this population, either because of thyroiditis, one of the most common autoimmune conditions, either because of pituitary disease in SAI. In this series 21 patients were taking a replacement therapy by l-Thyroxin for hypothyroidism, without any difference between PAI and SAI groups. Other treatments for hormonal insufficiency, in particular gonadotropic insufficiency, were reported by patients more often in the SAI group, which was expected. These co-morbidities can induce GI symptoms. We have then tested them in regression analyses in order to determine if they could explain GI symptoms. These variables were not risk factors for abdominal pain in our study. However, the questionnaire did not specifically address these issues and it is possible that some patients suffered from mild hormonal deficiency, which did not require replacement therapy but could have had an impact on digestive symptoms.

Another hypothesis is that digestive symptoms are related to impairment of the hypothalamic–pituitary–adrenal axis. Increased intestinal permeability has been described in several digestive diseases, such as inflammatory bowel disease (IBD) and IBS and several studies show that Corticotropin Releasing Hormone (CRH) plays a role in intestinal permeability regulation in rodents and in humans^14, 15^. IBS and IBD patients may also suffer of an impaired HPA axis. Indeed, while some IBS patients tend to have hypercortisolemia, IBD patients with prolonged systemic corticosteroid therapy tend to have hypocortisolemia^16, 17^. In this context, one could hypothesize that the loss of physiological secretion of cortisol or an inappropriate replacement therapy has an impact on intestinal permeability, even in treated patients, and therefore might induce digestive symptoms. However, to our knowledge, intestinal permeability has never been measured in CAI patients. An increased intestinal permeability could also modify cortisol absorption^18^, causing a vicious cycle. However, while abdominal pain and IBS-like symptoms were significantly higher in appropriate or inappropriate (under or overdosed) treatment subgroups as compared to controls, we did not find significant difference between appropriate or inappropriate treatment.

Autonomous nervous system (ANS) can also contribute to GI symptoms of CAI patients. Indeed, the corticotropic axis is connected to ANS which can also induce GI pain^19^. Beside the hypothesis that GI symptoms of our CAI patients are related to impairment of hypothalamic–pituitary–adrenal axis, these symptoms may then be explained by multifactorial elements.

Finally, patients reporting abdominal pain and altered bowel habits could suffer from an authentic IBS, without any connection to their adrenal insufficiency. Interestingly, the rate of previously diagnosed IBS in our series, 18%, is comparable to the rate of IBS in the European population estimated to be around 11.5%^20^. However, the rate of patients fulfilling IBS criteria is higher (30%) in our study. This result has to be discussed with caution, because the results of the Rome IV questionnaire are not sufficient to diagnose IBS without examination by a physician. However, it suggests that roughly a third of CAI patients complain about abdominal discomfort related to bowel habits. In our series, these symptoms appear to be related to all types of bowel habits among patients fulfilling IBS criteria, with maybe a predominance of IBS-M (34%) and IBS-U (34%). IBS-M is often described in the literature as one of the most frequent IBS subtypes^21^. The relatively high rate of patients fulfilling IBS-U criteria highlights the fact that these patients complain more about abdominal discomfort than about altered bowel habits. The predominance of female patients in this series probably plays a role in the reporting of digestive symptoms, as, indeed IBS is considered more frequent in women in the Western world^22^. In addition, digestive symptoms are not always a consequence of adrenal crisis, in fact acute infection particularly GI infection (1/5) are a frequent underlying cause of adrenal crises^2^.

We failed to identify risk factors for abdominal pain, apart from a former diagnosis of IBS, possibly because of a lack of statistical power. Similarly, digestive symptoms did not seem to have an impact on the patients’ quality of life, even though IBS is associated with impaired HRQoL^23^. There is a need for a case–control study on the subject of digestive symptoms in CAI patients.

There is increased awareness of the role of altered gut microbiota in a range of conditions, including in IBS^24^. Thyroid disorders, which are present in at least 17.6% of our patient group, are associated with increased prevalence of digestive disorders^25^ and gut-microbial disturbances^26^. The gut microbiome also influences the response of the pituitary and adrenal glands, as well as gut metabolism of medications. Analysis of gut microbiota should then be considered in future research on the various groups of CAI patients.

Although the Rome IV questionnaire assesses the full range of functional gastrointestinal disorders (FGIDs), we used only the section pertaining functional bowel. The shortcoming of limiting the research question to IBS (rather than FGIDs) in similar investigations in specific disease groups has been identified^27^. Is it possible that the patient cohort of interest suffers a disproportionate range of other gastrointestinal disorders.

Our study also highlights the poor or very poor quality of life described by CAI patients, whatever the group studied. The study design did not allow us to find an association between quality of life and digestive symptoms. However, we believe that the management of digestive symptoms should be considered in our efforts to improve the quality of life of CAI patients. Indeed, studies on IBS patients have demonstrated that the severity of GI symptoms is negatively associated with the “physical” dimension of health-related quality of life^28^ and that the patient quality of life depends on IBS subtype, diarrhea have lower disease specific quality of life than constipation^29^.

To conclude, our study shows that the prevalence of digestive symptoms in CAI patients may be very high. If this cross-sectional data is confirmed in studies with a higher population size, digestive symptoms should be taken into account in HRQoL questionnaires and more broadly, by physicians following CAI patients.

## Patients and methods

### Patients

From June to October 2017, an online bowel disorder questionnaire, entitled ‘chronic adrenal insufficiency and digestive symptoms’, adapted from the Rome IV classification^26^, was published on the website of the NPPA *“Adrenals”* a certified French non-profit patient association, partnered with the French National Authority for Health, supporting patients with adrenal disease. This study was performed at the Biology Department of Nantes University Hospital. No change in our current clinical practice nor randomization was performed. All methods were carried in accordance with relevant guidelines and regulations. According to French legislation (articles L.1121-1 paragraph 1 and R1121-2, Public health code), Ethics committee approval was not required for this study, as it uses data from HRQoL questionnaires. Anonymized data was used, which falls outside the scope of EU data protection law. Information about demographics and adrenal insufficiency (age at diagnosis, etiology, type of medication) was collected. Informed consent to participate in the study was obtained from all subjects before they completed the questionnaire.

Inclusion criteria were patients over 16, who had completed the questionnaire, and who had a history of treated adrenal insufficiency. Patients with a personal history of inflammatory bowel disease were excluded. The questionnaire was also submitted to CAI over 16 patients’ relatives (control group).

Completed questionnaires were first reviewed to eliminate duplicates. They were then analyzed by a gastroenterologist (LQ) and an endocrinologist (DD) in order to identify irrelevant and missing data.

The sample size of our CAI cohort was not estimated prior to the study. All groups were obtained after a time-to-respond on the publicized questionnaire.

### Digestive assessment

Information about history of digestive disease (a known history of irritable bowel syndrome (IBS), frequent episodes of gastroenteritis, etc.) and digestive symptoms, such as frequency of abdominal discomfort, connection between bowel habit and abdominal discomfort was collected. The consistence of stool was graded from 1 to 7 on the Bristol stool scale. Evaluation of digestive symptoms compatible with IBS as well as the classification of patients into subgroups of IBS was performed according to the Rome IV criteria^30^. Patients were also asked to quantify their quality of life using a 5-point scale from ‘1-very poor’ to ‘5-excellent’.

### Assessment of endocrine parameters

Information about type and duration of CAI was collected. The type of substitution treatment as well as the posology was noted. Treatment was considered to be appropriately dosed at 20–30 mg hydrocortisone per day for PAI, 10–20 mg hydrocortisone per day for SAI, and 20–40 mg hydrocortisone per day for CAH. Treatment was considered to be inappropriate if the dosages were lower or higher. As Dexamethasone, a synthetic glucocorticoid, has a long plasma half-life and longer duration of action compared to hydrocortisone, for those on dexamethasone, treatment was also considered to be inappropriate.

### Statistical analysis

Analysis was performed using SAS version 9.4 (Cary, NC, USA). Patients were divided in 3 groups according to the etiology of CAI. Categorical variables were expressed as percentages and quantitative variables as mean and standard deviation. Comparisons across groups and control group were made using Chi2 test, or Fisher exact test, for categorical data and with ANOVA test for quantitative data. Pairwise multiple comparisons were made with Tukey–Kramer's method for quantitative variables and Chi2 test, or Fisher exact test, for categorical variables with Holm correction. An analysis of risk factors of abdominal pain was performed using univariate and multivariate logistic regression. The multivariate model was constructed using backward method from variables with a *p* value < 0.20 in the univariate analysis. The adequacy of the model was verified using Hosmer and Lemeshow's test. The level of significance was set at *p* < 0.05.
